# Correction to: The Room

**DOI:** 10.1007/s10912-024-09910-9

**Published:** 2024-11-05

**Authors:** Virginjia Vilkelyte, Luna Dolezal, Juanita Navarro-Páez, Charlotte A. Wu, Will Bynum, Zara Slattery

**Affiliations:** 1 University of Exeter Medical School, Heavitree Road, Exeter, EX1 2LU UK; 2 Wellcome Centre for Cultures and Environments of Health (WCCEH), University of Exeter, Queen’s Building, Queen’s Drive, Exeter, EX4 4QH UK; 3 Global Business School for Health and Harness Health Global, University College London, Lombard House, Cross Keys, Lichfield, WS13 6DN UK; 4 Duke University School of Medicine, 2100 Erwin Rd, Durham, NC 27705 USA; 5 Brighton, UK

**Correction to: Journal of Medical Humanities**

10.1007/s10912-024-09899-1

In the recently published article, the funding note below should be added to the proof.

Funding The study was funded by the Wellcome Trust (217879/Z/19/Z), Duke-Exeter Accelerator Grant and Wellcome Centre for Cultures and Environments of Health Enhanced Research Award.

The original article has been corrected.

